# Predictive value of RAD51 on the survival and drug responsiveness of ovarian cancer

**DOI:** 10.1186/s12935-021-01953-5

**Published:** 2021-05-05

**Authors:** Yuchen Feng, Daoqi Wang, Luyang Xiong, Guohua Zhen, Jiahong Tan

**Affiliations:** 1 Division of Respiratory and Critical Care Medicine, Department of Internal Medicine, Tongji Hospital, Tongji Medical College, Huazhong University of Science and Technology, Wuhan, 430030 People’s Republic of China; 2 Department of Urology, Tongji Hospital, Tongji Medical College, Huazhong University of Science and Technology, Wuhan, 430030 People’s Republic of China; 3 Department of Emergency, Tongji Hospital, Tongji Medical College, Huazhong University of Science and Technology, Wuhan, 430030 People’s Republic of China; 4 Department of Obstetrics and Gynecology, Tongji Hospital, Tongji Medical College, Huazhong University of Science and Technology, Wuhan, 430030 People’s Republic of China

**Keywords:** Drug responsiveness, Ovarian cancer, Predictor, RAD51, Survival

## Abstract

**Background:**

Ovarian cancer has greatly endangered and deteriorated female health conditions worldwide. Refinement of predictive biomarkers could enable patient stratification and help optimize disease management.

**Methods:**

RAD51 expression profile, target-disease associations, and fitness scores of RAD51 were analyzed in ovarian cancer using bioinformatic analysis. To further identify its role, gene enrichment analysis was performed, and a regulatory network was constructed. Survival analysis and drug sensitivity assay were performed to evaluate the effect of RAD51 expression on ovarian cancer prognosis. The predictive value of RAD51 was then confirmed in a validation cohort immunohistochemically.

**Results:**

Ovarian cancer expressed more RAD51 than normal ovary. RAD51 conferred ovarian cancer dependency and was associated with ovarian cancer. RAD51 had extensive target-disease associations with various diseases, including ovarian cancer. Genes that correlate with and interact with RAD51 were involved in DNA damage repair and drug responsiveness. High RAD51 expression indicated unfavorable survival outcomes and resistance to platinum, taxane, and PARP inhibitors in ovarian cancer. In the validation cohort (126 patients), high RAD51 expression indicated platinum resistance, and platinum-resistant patients expressed more RAD51. Patients with high RAD51 expression had shorter OS (HR = 2.968, P < 0.0001) and poorer PFS (HR = 2.838, P < 0.0001). RAD51 expression level was negatively correlated with patients’ survival length.

**Conclusions:**

Ovarian cancer had pronounced RAD51 expression and RAD51 conferred ovarian cancer dependency. High RAD51 expression indicated poor survival and decreased drug sensitivity. RAD51 has predictive value in ovarian cancer and can be exploited as a predictive biomarker.

**Supplementary Information:**

The online version contains supplementary material available at 10.1186/s12935-021-01953-5.

## Background

Among the malignancies threatening women’s health, ovarian cancer is the most lethal and represents a salient public health concern [1–3]. Epithelial ovarian cancer comprises 95% of ovarian cancer and contributes to the death of more 230,000 women worldwide annually [4–6]. In the late 1970s, cisplatin arose as a therapeutic regimen for ovarian cancer patients [7, 8]. The late 1980s witnessed the introduction of carboplatin and taxane [7, 8]. Since 1996, the landmark combination regimen of cisplatin and paclitaxel following surgical cytoreduction has been a mainstay in the treatment of ovarian cancer [7, 8]. Aiming to specifically kill cancer cells, targeted therapy has ushered a new era for ovarian cancer treatment [4, 5]. Poly-(ADP-ribose)-polymerase (PARP) inhibitors display promising antitumor effects by leveraging synthetic lethality and ameliorate some concerns in handling ovarian cancer [9]. Despite steady advance in research, the disconcerting situation faced by ovarian cancer patients has persisted, and the 5-year survival rate has remained at approximately 40% for decades [1, 8]. Realization of individualized precision medicine requires refinement of predictive biomarkers, which would enable patient stratification and selection and could help optimize disease management [4, 10].

Ovarian cancer shows a predisposition to genomic instability, which is a pervasive characteristic feature of most solid tumors [11]. The devastating genomic instability drives disease progression and the potential for drug resistance but also highlights the possibility for treatment [12]. Defects in cancer genome have already been recognized in many commonly used treatment regimens [13]. For example, platinum agents induce DNA inter- and intrastrand crosslink lesions, which cannot be repaired efficiently by ovarian cancer cells with impaired genomes [11]. DNA damage repair acts as a brake and protects against genomic instability and can affect the response to DNA-damaging anticancer therapy [11, 12]. DNA damage repair pathways could be exploited as ideal targets for therapeutic intervention [11, 12]. The TCGA network has allowed for a characterization of the genetic signatures of ovarian cancer by integrated genomic analysis, laying the foundation for the discovery of potential predictive biomarkers [14].

RAD51 or RAD51 recombinase, also known as RAD51A, which catalyzes the recognition of homology and strand exchange and enables timely DNA damage repair, is essential for the maintenance of genome integrity [15]. RAD51 has a multifaceted role in carcinogenesis, cancer progression, and anticancer drug resistance [15, 16]. Epithelial–mesenchymal transition-associated drug resistance, hypoxia-mediated drug tolerance, and drug resistance in cancer stem cells all involve RAD51 [15, 16]. RAD51 can also upregulate prometastatic gene expression [16]. RAD51 is a crucial component and functions at the core of homologous recombination, which recruits and allocates various mediators or regulators or interactors, such as BRCA2, PALB2, and TOPBP1 [15, 16]. RAD51 overexpression leads to excessive and improper recombination, thereby triggering genomic instability, which successively drives malignant transformation, contributes to tumor progression, and even induces anticancer drug tolerance [15–17]. Under exposure to exogenous insults, formation of RAD51 foci in cell nucleus represents the assembly and initiation of DNA damage repair [18]. Recently, RAD51 was reported to be a reliable surrogate marker capable of predicting the capacity for homologous recombination repair [18, 19].

Here, we explored the predictive value of RAD51 in ovarian cancer. RAD51 expression in cancers was profiled to get a general insight and its expression in ovarian cancer was then studied in detail. The target-disease associations of RAD51 were assessed to evaluate its role in ovarian cancer. Genes that correlate with or interact with RAD51 were further analyzed. The effect of RAD51 expression on survival and drug responsiveness in ovarian cancer was examined in online databases. Finally, the predictive value of RAD51 was confirmed in the validation phase.

## Methods

### cBioPortal

As an open-source resource for interactive exploration of multidimensional cancer genomics datasets, cBioPortal (https://www.cbioportal.org) provides high-quality access to molecular profiles and clinical attributes from large-scale cancer genomic projects [20]. By exploring cBioPortal, we obtained the mutation profile and expression patterns of RAD51. In addition, clinicopathological characteristics of involved patients were also retrieved from cBioPortal. The effect of RAD51 expression on disease-free months of ovarian cancer patients was evaluated in survival analysis, and log-rank test was used.

### Gene Expression Profiling Interactive Analysis (GEPIA)

To facilitate data mining and a deeper understanding of gene functions, GEPIA (http://gepia.cancer-pku.cn) delivers fast and customizable functionalities based on TCGA and GTEx data [21]. The profile plotting and comparison of RAD51 expression between various tumor tissues and matched normal tissues were performed in GEPIA using one-way ANOVA test. Correlation analysis concerning RAD51 was implemented using GEPIA, and Pearson’s correlation coefficient was calculated.

### The Human Protein Atlas

Aiming to map all human proteins in cells, tissues, and organs, the Human Protein Atlas (https://www.proteinatlas.org) has created a genome-wide map of the human proteome with integration of various omics technologies [22]. We assessed RAD51 expression in the major tissues and organs by using the Human Protein Atlas.

### Kaplan–Meier plotter

Integrating gene expression data and clinical information from GEO, EGA, and TCGA, Kaplan–Meier plotter (https://kmplot.com/analysis/) can analyze the prognostic value of a particular gene, which enables discovery and validation of survival biomarkers [23]. Ovarian cancer patients were divided into two groups based on RAD51 expression. Using Kaplan–Meier plotter, the survival probability of the two cohorts was compared and the hazard ratio (HR) with 95% confidence intervals and log-rank P value were calculated.

### UALCAN

UALCAN (http://ualcan.path.uab.edu), a comprehensive and interactive web resource, promotes gene-level queries of TCGA data and accelerates cancer research [24]. We searched UALCAN for genes correlated with RAD51 in ovarian cancer.

### Open Targets platform

For systemic drug target identification and prioritization, the Open Targets platform (https://www.targetvalidation.org) collects public domain data of human genetics and genomics [25]. Using the Open Targets platform, we evaluated and scored the target-disease associations of RAD51. The top 20 targets related to RAD51 were also retrieved from the Open Targets platform.

### STRING

The online web portal STRING (https://string-db.org) achieves a comprehensive and objective global network of protein–protein interactions, including physical and functional interactions [26]. The protein–protein interaction network of RAD51 was constructed and visualized using STRING.

### Project Score database

Project Score database (https://score.depmap.sanger.ac.uk) uses genome-scale CRISPR–Cas9 screens to identify dependencies operative in all cancers [27]. A total of 323 cell lines from 19 tissues and 7470 fitness genes are archived in Project Score database, which provides insights into basic cancer biology and helps guide precision cancer medicine. In Project Score database, the fitness scores of RAD51 in ovarian cancer lines were examined.

### Oncomine

Oncomine (https://www.oncomine.org/resource/main.html) is a systematic data-mining platform comprising 48 million gene expression measurements from over 4700 microarray experiments [28]. Differential expression of RAD51 was explored and queried across a panel of ovarian cancer analyses in Oncomine.

### R2

R2: Genomics Analysis and Visualization Platform (http://r2.amc.nl) is a biologist-friendly web-based application of genomics analysis and visualization. Taking advantage of R2, we determined gene expression of TCGA ovarian cancers.

### Cancer Dependency Map (DepMap)

Genes that are essential for cancer cell survival delineate cancer dependencies. DepMap reinforces the understanding of the relationship between the genetic alterations and the resulting cancer dependencies, thereby promoting identification of genetic and pharmacologic dependencies and the biomarkers that predict them [29]. Using DepMap, the mutual relation between RAD51 gene effect and drug sensitivity of ovarian cancer cell lines was evaluated using Pearson’s correlation test.

### TIMER analysis

The webserver TIMER2.0 (http://timer.cistrome.org) provides comprehensive analysis and visualization of immune infiltrates across diverse cancer types [30]. TIMER database was used to evaluate the effect of RAD51 expression on tumor-infiltrating immune cells in ovarian cancer using Spearman’s correlation test.

### Clinical samples

A total of 126 high-grade serous ovarian cancer patients from the Gynecology Oncology Department of Tongji Hospital, Tongji Medical College, Huazhong University of Science and Technology were included. With signed informed consent, all clinical samples were collected at initial cytoreductive surgery without preoperative adjuvant chemotherapy or radiotherapy. This study was approved by the Ethics Committee of Tongji Medical College, Huazhong University of Science and Technology. During follow-up, serological tests for CA125 and radiological examinations, usually B-ultrasound, were conducted. The follow-up duration lasted from January 2014 to January 2021 with a median range of 48 months. All patients have received debulking surgery followed by platinum-taxane chemotherapy. Within 6 months after therapy, platinum-resistant tumor progressed, while platinum-sensitive cancer did not recur or relapse [7].

### Immunohistochemistry

Immunohistochemical analysis was performed as reported previously [31]. Tumor slides were subjected to heat-induced antigen retrieval using citrate buffer (pH 6.0, G1202, ServiceBio, China). An Avidin–Biotin Complex Vectastain Kit (SP-9001, Zsgb-Bio, China) was used according to the manufacturer’s guidelines for the following procedures. The primary antibody used was anti-RAD51 (14961-1-AP, Proteintech, China). The HSCORE immunoreactivity scoring system was used to quantify RAD51 expression. Briefly, *i* depicts the staining intensity of tumor cells, *Pi* means the percentage of cells at the corresponding intensity, and HSCORE = ∑ *i* × *Pi*. Two investigators scored all slides and were blinded to patient characteristics. HSCORE ≤ 1.5 was designated as low protein expression, and HSCORE > 1.5 was classified as high RAD51 expression.

### Statistical analysis

Statistical analyses of bioinformatics were described above. To evaluate categorical variables, Chi-squared test was used. A two-sided Student’s *t*-test was performed to compare RAD51 expression between the platinum-sensitive group and platinum-resistant group. For survival analysis, log-rank test was employed. Pearson’s correlation test was implemented in correlation analysis. To detect the correlation between RAD51 expression and clinicopathological variables, Fisher’s exact test or Chi-squared test for trend were performed. GraphPad Prism 7 (GraphPad Software, Canada) was used to analyze and present data (mean ± SD). Significance was assessed at the level of P < 0.05.

## Results

### Expression profile of RAD51

**Fig. 1:**
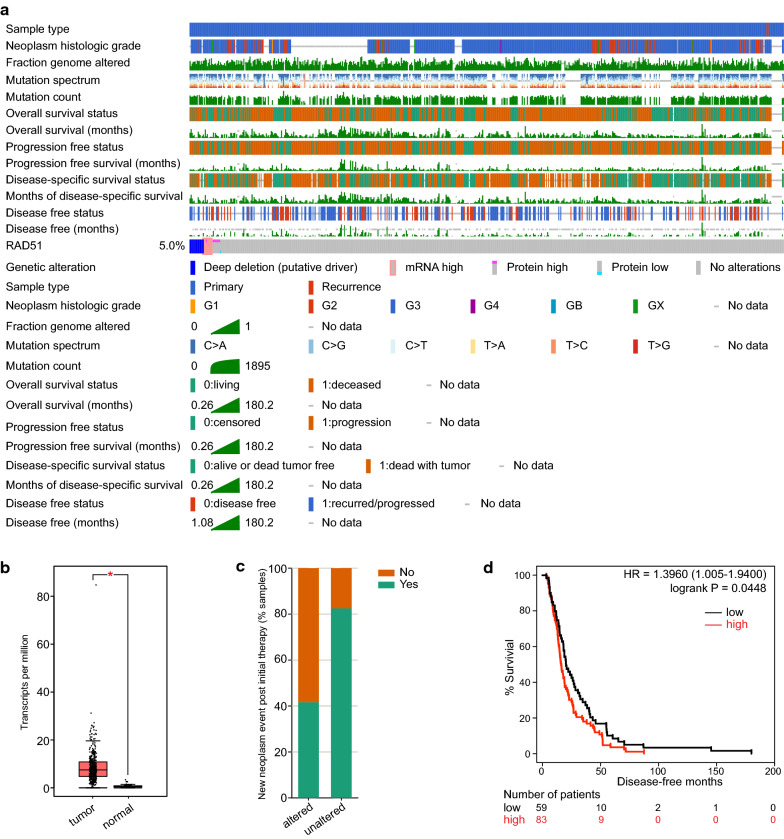
Expression profile of RAD51 in ovarian cancer. **a** Clinicopathological characteristics of TCGA ovarian cancer patients and genetic signatures of RAD51 were studied in detail and depicted as oncoprints using cBioPortal. **b** Comparison of RAD51 expression between TCGA ovarian cancer tissues and matched GTEx normal tissues in GEPIA (one-way ANOVA). **c** Proportion of patients who developed new neoplasm events post initial therapy was analyzed in cBioPortal. **d** The effect of RAD51 expression on disease-free months of ovarian cancer patients was evaluated and survival analysis was performed (log-rank test). P value was denoted as *P < 0.05

To comprehensively characterize RAD51, we searched the Human Protein Atlas and obtained its general expression in various tissues and organs (Additional file 1: Fig. S1a). Normal ovary had a medium RAD51 expression. When comparing tumor samples and pair normal samples, 19 kinds of tumor subtypes had elevated RAD51 expression (19/33), and one kind had significantly decreased RAD51 expression (1/33) (Additional file 1: Fig. S1b). By exploring cBioPortal, we retrieved the mutation profile of RAD51 in individual TCGA cancer and in a collection of TCGA cancers (Additional file 1: Fig. S1c, Additional file 2: Fig. S2). Deep deletion was the most common genetic alteration of RAD51 observed in ovarian cancer. To further profile RAD51 in ovarian cancer, the genetic signatures and genetic alterations found in TCGA ovarian cancers were analyzed in detail (Fig. 1a, Additional file 3: Fig. S3a). Ovarian cancer tissues had pronounced RAD51 expression compared with paired normal tissues (Fig. 1b). More patients with unaltered RAD51 had experienced new neoplasm events post initial therapy, suggesting a possibly deleterious role of RAD51 in ovarian cancer (Fig. 1c). Patients with definite disease-free status were then split into a disease-free group and a recurred/progressed group. Although the two groups had nearly the same RAD51 expression, patients with high RAD51 expression had shorter disease-free survival times (HR = 1.3960, P = 0.0448) (Fig. 1d, Additional file 3: Fig. S3b). Together, ovarian cancer expresses more RAD51 than normal ovary, and RAD51 affects the prognosis of ovarian cancer patients.

### RAD51 confers cancer dependency in ovarian cancer

**Fig. 2:**
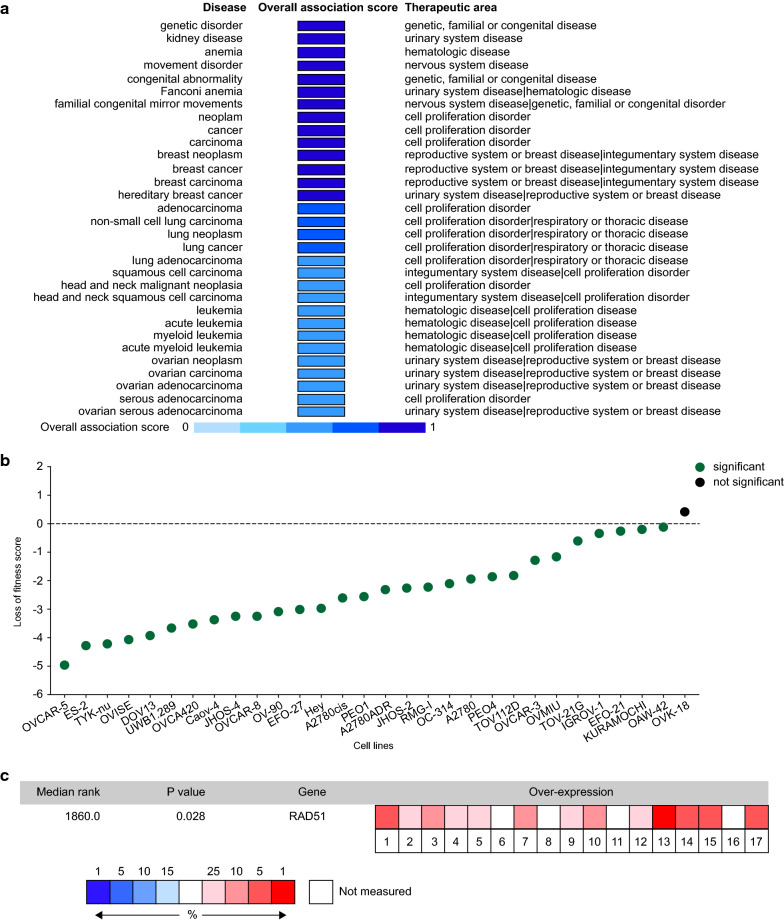
RAD51 confers ovarian cancer dependency. **a** Using the Open Targets platform, target-disease associations of RAD51 were calculated, and those scoring higher than 0.5 were depicted. **b** Fitness score is a quantitative evaluation of the cell viability effect elicited by CRISPR–Cas9-mediated cell inactivation. In Project Score Database, the fitness scores of RAD51 in 30 ovarian cancer cell lines were explored. **c** Integrated analysis of RAD51 expression in 17 datasets using Oncomine

Integrating information concerning RAD51, including genetic associations, somatic mutations, drugs, pathways and system biology, RNA expression, text mining, and animal models, target-disease associations of RAD51 were calculated. Among the 31 diseases associated with RAD51, which scored higher than 0.5, ovarian neoplasm, ovarian carcinoma, ovarian adenocarcinoma, and ovarian serous adenocarcinoma were confirmed (Fig. 2a). In Project Score Database, fitness score is a quantitative evaluation of the cell viability effect elicited by CRISPR–Cas9-mediated cell inactivation. In the 30 ovarian cancer cell lines archived, only one did not have a statistically significant score (Fig. 2b). We then examined RAD51 expression in 17 datasets using Oncomine, and RAD51 was found to be significantly overexpressed in ovarian cancers (P = 0.028) (Fig. 2c). Overall, RAD51 mediates ovarian cancer dependency.

### RAD51 is associated with ovarian cancer

Nine genes were reported to be significantly mutated genes in ovarian cancer, namely, BRCA1, BRCA2, CDK12, CSMD3, FAT3, GABRA6, NF1, RB1, and TP53 [14]. Except for CSMD3, FAT3, and GABRA6, the other six genes were significantly positively correlated with RAD51 (Additional file 4: Fig. S4a). Their expression was also profiled in TCGA ovarian cancers (Additional file 4: Fig. S4b).

**Fig. 3:**
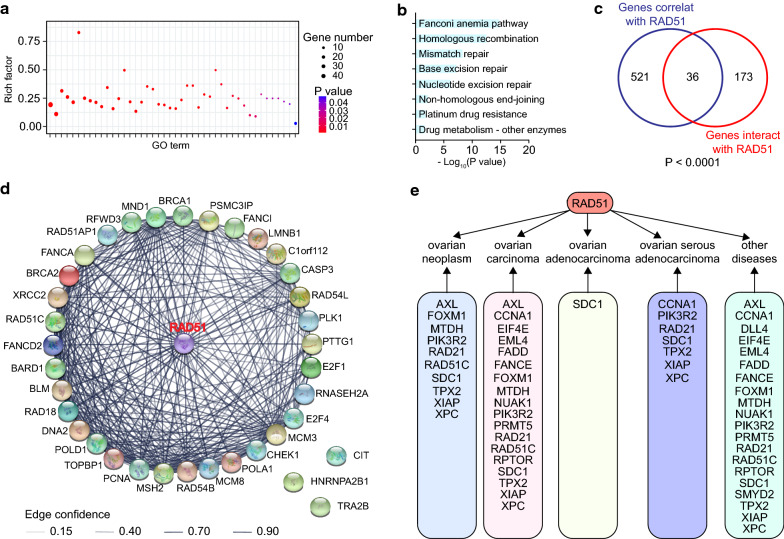
RAD51 associates with ovarian cancer. In UALCAN, 557 genes correlated with RAD51 in TCGA ovarian cancer. Enrichment analysis was performed using these 557 genes. **a** Gene Ontology pathways involved in DNA damage repair and drug responsiveness were shown. **b** KEGG pathways implicated in DNA damage repair and drug responsiveness were depicted. In NCBI, 209 genes interacted with RAD51. These 209 genes overlapped with the 557 genes correlated with RAD51, producing 36 overlapping genes. **c** Venn diagram of these 209 genes and the 557 genes correlated with RAD51 (Chi-squared test). **d** The protein–protein interaction network of RAD51 with the 36 overlapping genes was constructed, and interaction confidence was calculated. **e** Based on computation of similar target-to-disease connections and overall association scores, the targets related to RAD51 were examined in the Open Targets platform and the top 20 targets were depicted in detail

After searching UALCAN, we obtained 557 genes correlated with RAD51 in TCGA ovarian cancer (Additional file 5: Table S1). In Gene Ontology analysis, these 557 genes were significantly enriched in 44 pathways related to DNA damage repair and drug responsiveness, such as interstrand cross-link repair, double-strand break repair, and response to drug (Fig. 3a, Additional file 6: Table S2). In KEGG enrichment analysis of the 557 genes, eight pathways implicating in DNA damage repair and drug responsiveness were significantly affected by RAD51, including homologous recombination, platinum drug resistance, and drug metabolism (Fig. 3b, Additional file 7: Table S3). By exploring the NCBI database, we identified 209 genes that interact with RAD51 (Additional file 8: Table S4). These 209 genes overlapped with the 557 genes correlated with RAD51 (P < 0.0001) (Fig. 3c). The 36 overlapping genes were analyzed and visualized using STRING (Fig. 3d, Additional file 9: Fig. S5). Some genes associated with DNA damage repair and drug sensitivity had direct protein–protein interactions with RAD51, for example, BRCA1, BRCA2, TOPBP1, and CHEK1, forming a regulatory network. Based on computation of similar target-to-disease connections and overall association scores, we examined the targets related to RAD51 in the Open Targets platform, and the top 20 targets were studied in detail (Fig. 3e). 20 genes represented targets related to RAD51 in disease conditions, including ten in ovarian neoplasm, 18 in ovarian carcinoma, one in ovarian adenocarcinoma, and seven in ovarian serous adenocarcinoma. In summary, RAD51 has broad connections with ovarian cancer.

### High RAD51 expression denotes poor survival outcomes in ovarian cancer

**Fig. 4:**
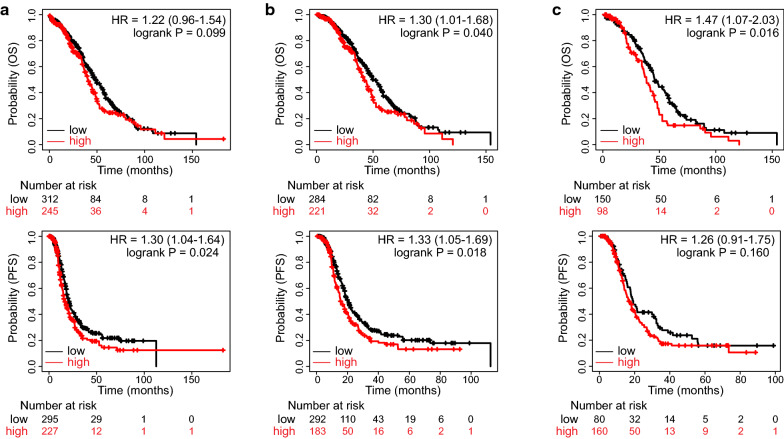
High RAD51 expression denotes unfavorable survival in TCGA ovarian cancer. According to RAD51 expression, patients in the TCGA dataset were split into two groups. Survival analysis was performed and Kaplan–Meier survival curves of OS and PFS in **a** the general patients, **b** patients receiving platinum-containing chemotherapy, and **c** patients treated with taxol were shown (log-rank test)

Accordingly, RAD51 might affect the prognosis of ovarian cancer. Survival analyses were performed in the TCGA dataset (Fig. 4a). Although the difference in overall survival (OS) did not reach the predefined significance threshold, patients with high RAD51 expression tended to have decreased OS (HR = 1.22, P = 0.099). High RAD51 expression represented a risk factor for progression-free survival (PFS) (HR = 1.30, P = 0.024). In patients receiving platinum-based chemotherapy, high RAD51 expression indicated shorter OS and PFS times (Fig. 4b). When treated with taxol, patients with high RAD51 expression had worse OS and were inclined to have reduced PFS time (Fig. 4c).

Consistently, high RAD51 expression deteriorated survival outcomes of ovarian cancer patients in GSE26193 (OS, HR = 2.04, P = 0.013; PFS, HR = 1.91, P = 0.023) (Additional file 10: Fig. S6a). In patients having platinum-containing treatment regimens, high RAD51 expression conferred disadvantages for OS and PFS (Additional file 10: Fig. S6b). There was no statistically significant survival difference concerning RAD51 in taxol-treated patients, yet the RAD51 high proportion exhibited a poorer survival outcome (Additional file 10: Fig. S6c). Similar results were obtained in GSE63885 (Additional file 11: Fig. S7). High RAD51 expression predicted unfavorable OS in all patients (HR = 1.79, P = 0.020), in platinum-treated patients (HR = 1.79, P = 0.020), and in taxol-exposed patients (HR = 5.23, P = 3e-5). In general patients and in platinum-treated patients in the GSE63885 dataset, RAD51 negatively affected PFS. In patients receiving taxol-containing treatment, high RAD51 expression implied shorter PFS times (HR = 2.78, P = 0.0054). These results suggested that high RAD51 expression indicates poor prognosis in ovarian cancer.

### High RAD51 expression indicates increased drug tolerance in ovarian cancer

**Fig. 5:**
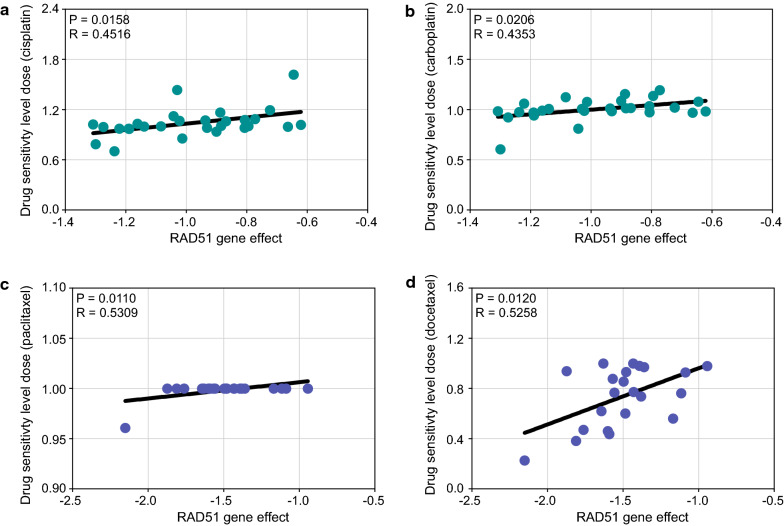
High RAD51 expression indicates increased chemoresistance in ovarian cancer. In DepMap, drug sensitivity level dose was used to evaluate drug sensitivity, namely, the higher the drug sensitivity level dose was, the more resistant the cell line. Correlation analysis between RAD51 gene effect and drug sensitivity level dose of ovarian cancer lines to **a** cisplatin, **b** carboplatin, **c** paclitaxel, and **d** docetaxel was performed (Pearson’s correlation test)

Subsequently, we examined the effect of RAD51 on drug responsiveness of ovarian cancer cell lines. Drug sensitivity level dose was used to evaluate drug sensitivity; namely, the higher the drug sensitivity level dose was, the more resistant the cell line. In DepMap, RAD51 gene effect positively correlated with platinum sensitivity level dose (cisplatin, P = 0.0158, R = 0.4516; carboplatin, P = 0.0206, R = 0.4353), suggesting that RAD51 mediates platinum resistance (Fig. 5a, b). Similarly, RAD51 mediated paclitaxel resistance (P = 0.0110, R = 0.5309) and docetaxel tolerance (P = 0.0120, R = 0.5258) (Fig. 5c, d).

PARP inhibitors now bring benefits to an increasing number of ovarian cancer patients [4]. Notably, RAD51 affected PARP inhibitor responsiveness of ovarian cancer cell lines (Additional file 12: Fig. S8). In olaparib, niraparib, and talazoparib, there was a positive correlation between RAD51 gene effect and PARP inhibitor sensitivity level dose (olaparib, P = 0.0475, R = 0.4270; niraparib, P = 0.0377, R = 0.4357; talazoparib, P = 0.0137, R = 0.5173). For rucaparib, high RAD51 gene effect reduced rucaparib sensitivity (P = 0.0335, R = − 0.4892). Furthermore, RAD51 expression affected immune infiltration in ovarian cancer (Additional file 13: Fig. S9). Overexpressed RAD51 significantly correlated with reduced B cell, T cell, and hematopoietic stem cell infiltration, but increased myeloid-derived suppressor cell infiltration, indicating a suppressive immune microenvironment and inclination to resist immune therapy. Taken together, RAD51 confers drug tolerance, including platinum, taxane, and PARP inhibitors, in ovarian cancer.

### RAD51 expression predicts platinum resistance and poor survival in ovarian cancer

**Fig. 6:**
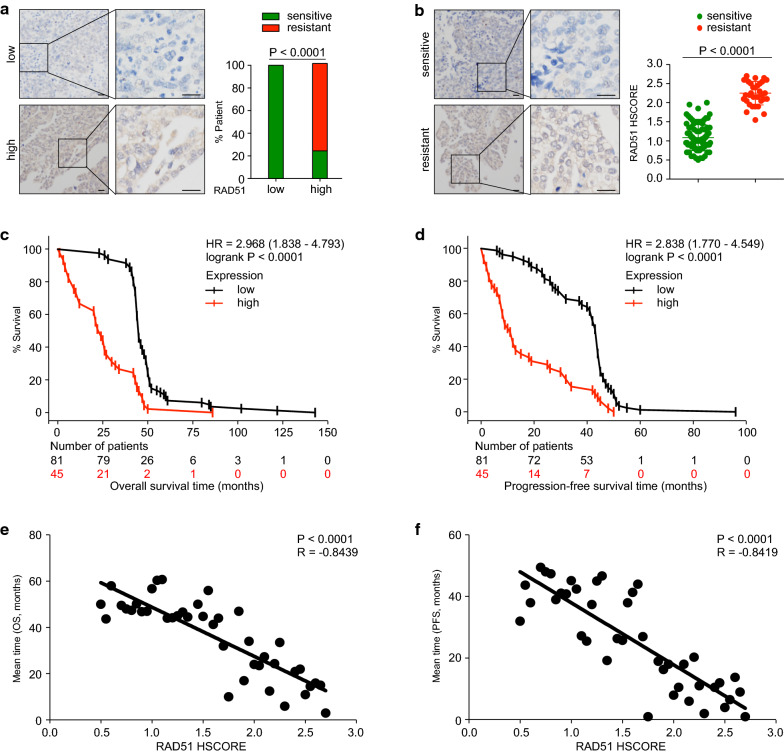
RAD51 expression predicts platinum sensitivity and survival of ovarian cancer. RAD51 expression was detected immunohistochemically in a cohort of 126 patients and quantified using HSCORE. The patients were subdivided into two groups: HSCORE ≤ 1.5, low expression; HSCORE > 1.5, high expression. Platinum sensitivity was defined as no recurrence within 6 months after therapy, while platinum resistance represented recurrence or relapse within 6 months. **a** Platinum responsiveness of ovarian cancer patients concerning RAD51 expression was analyzed (Chi-squared test). **b** Comparison of RAD51 expression between the platinum-sensitive group (N = 92) and the platinum-resistant group (N = 34) (Student’s *t*-test). Survival analysis was performed and Kaplan–Meier survival curves for OS (**c**) and PFS (**d**) were depicted (log-rank test). Correlation analysis between RAD51 expression and mean survival time of patients with the same HSCORE was performed for OS (**e**) and PFS (**f**) (Pearson’s correlation test)

To further confirm the role of RAD51 in ovarian cancer, we detected RAD51 immunohistochemically in a cohort encompassing 126 ovarian cancer patients. Characteristics of included patients were summarized in Table 1. Platinum sensitivity was defined as no progression/recurrence within six months after platinum-taxane chemotherapy [7]. More patients with high RAD51 expression (HSCORE > 1.5) had platinum-resistant disease (Fig. 6a). Comparison of RAD51 expression between the platinum-sensitive group (N = 92) and the platinum-resistant counterpart (N = 34) produced consistent results (Fig. 6b). Patients having platinum-resistant disease expressed significantly higher levels of RAD51 (P < 0.0001). By survival analysis, patients with high RAD51 expression had shorter OS (HR = 2.968, P < 0.0001) and poorer PFS (HR = 2.838, P < 0.0001) (Fig. 6c, d). We then analyzed the correlation between HSCORE, representing RAD51 expression, and the mean survival time of patients with the same HSCORE. RAD51 expression negatively correlated with OS months (P < 0.0001, R = − 0.8439) and PFS months (P < 0.0001, R = − 0.8419). Therefore, high RAD51 expression indicates platinum resistance and unfavorable survival in ovarian cancer patients.

**Table 1:** Summary of patient characteristics. FIGO, International Federation of Gynecology and Obstetrics

Characteristics	Total patients	RAD51 low		RAD51 high		*P* values
	(N = 126)	(N = 81)		(N = 45)		
	No	No	%	No	%	
Histology	High-grade serous	High-grade serous		High-grade serous		
Age at diagnosis (years)						
≤ 50	53	36	44.44%	17	37.78%	0.5726^a^
> 50	73	45	55.56%	28	62.22%	
FIGO stage						
I	9	6	7.41%	3	6.67%	0.5332^b^
II	22	15	18.52%	7	15.56%	
III	84	54	66.66%	30	66.66%	
IV	11	6	7.41%	5	11.11%	
Ascites						
Yes	81	53	65.43%	28	62.22%	0.4916^b^
No	25	17	20.99%	8	17.78%	
Unknown	20	11	13.58%	9	20.00%	

^a^Fisher’s exact test

^b^Chi-squared test for trend

## Discussion

Discovery of biomarkers for ovarian cancer is of great importance [10]. In the present study, we profiled the role of RAD51 in ovarian cancer. Ovarian cancer tissues expressed more RAD51 than normal ovary tissues. RAD51 had target-disease associations with ovarian cancer and conferred ovarian cancer dependency. By forming a complex regulatory network, RAD51 closely associated with ovarian cancer. Moreover, RAD51 affected DNA damage repair and drug responsiveness in ovarian cancer. High RAD51 expression predicted poorer survival in ovarian cancer patients and indicated drug tolerance, including platinum, taxane, and PARP inhibitors. Therefore, RAD51 has predictive value in ovarian cancer and could be exploited as a predictive biomarker for survival and drug responsiveness.

RAD51 was reported to be implicated in various cancers, such as prostate cancer, lung cancer, and breast cancer [15, 16]. Here, we found pronounced RAD51 expression in 19 kinds of cancers, implying that RAD51 might mainly function as an oncogene. In ovarian cancer, RAD51 expression was significantly elevated. Ovarian cancer patients with normal RAD51 gene were inclined to experience new neoplasm events post initial therapy. High RAD51 expression correlated with shorter disease-free survival time. The unfavorable role of RAD51 was further supported by the general target-disease associations of RAD51 with ovarian cancer. As a significantly overexpressed fitness gene, RAD51 conferred ovarian cancer dependency. These results suggest that RAD51 plays a pivotal role in ovarian malignancies. The top 20 targets related to RAD51 and RAD51 were all implicated in ovarian cancer. RAD51 overexpression usually results in treatment resistance and lower survival rates of patients [15, 16]. Specific inhibitors or antibodies for RAD51 have been developed to pharmacologically target RAD51, including B02, DIDS, and 3E10 [32–35]. Although RAD51-targeted therapies are still in the preclinical study or clinical trial phase, it is reasonable to believe that RAD51 is potentially a potent therapeutic target in ovarian cancer.

As an important executor of DNA damage repair, RAD51 principally participates in homologous recombination and can stabilize the replication fork [11, 16]. Elevated RAD51 expression increases homologous recombination competency of cancer cells and contributes to drug resistance to DNA-damaging chemotherapies [11, 15, 16]. By enrichment analysis, 557 genes correlated with RAD51 in TCGA ovarian cancer were significantly accumulated in Gene Ontology and KEGG pathways involved in DNA damage repair and drug responsiveness. Platinum agents exert anticancer effects by inducing interstrand cross-links, which requires RAD51 for repair [11, 16]. DNA double-strand breaks are extremely cytotoxic and rely on RAD51 for restoration [16, 36]. Besides, two KEGG pathways, namely, platinum drug resistance and drug metabolism, were directly affected by RAD51. RAD51 significantly correlated with six highly mutated genes in ovarian cancer and formed a regulatory network by protein–protein interactions, thereby being involved in ovarian tumorigenesis and associated with ovarian cancer. BRCA1 and BRCA2, both of which help maintain genomic stability and are significantly mutated in ovarian cancer, directly interact with RAD51. Whether in the general patients, or in patients receiving platinum-based chemotherapy, or in taxol-treated patients, high RAD51 expression deteriorated survival prognosis of ovarian cancer. Increased RAD51 expression remarkably correlated with reduced drug responsiveness to platinum, taxane, and PARP inhibitors in ovarian cancer. Overexpressed RAD51 promoted drug resistance to cisplatin and carboplatin. Increased RAD51 reduced drug sensitivity to paclitaxel and docetaxel. For the three approved PARP inhibitors olaparib, niraparib, and rucaparib, as well as the intensively studied PARP inhibitor talazoparib, elevated RAD51 expression denoted decreased drug responsiveness. In our validation cohort, high RAD51 expression indicated platinum resistance in ovarian cancer patients, and platinum-sensitive cancers expressed significantly less RAD51. High expression of RAD51 predicted unfavorable survival, and RAD51 expression level correlated with patient survival length. Therefore, RAD51 is a feasible biomarker for predicting drug responsiveness and survival in ovarian cancer. However, nearly 20% of patients with high RAD51 expression showed platinum sensitivity, necessitating further refinement of the expression threshold. Owing to its multifaceted roles in various cancers, RAD51 might have universal predictive value and warrants further investigation. Since RAD51 can be detected conveniently by immunohistochemistry, it might be also capable of tracking drug sensitivity during treatment.

High-grade serous ovarian cancer, accounting for 70% of epithelial ovarian cancer, predominates in clinical practice [4, 5]. The TCGA ovarian cancer dataset consisted of serous ovarian cancer [14]. In the validation phase, the recruited patients had high-grade serous ovarian cancer. The role of RAD51 in other ovarian cancer subtypes needs further exploration. In clinical treatment schedules, platinum and taxane are generally used in combination. Owing to the retrospective nature of the present study, we were limited to determining the effect of RAD51 on platinum treatment or taxane therapy and could only assess platinum sensitivity. Another limitation was that we did not have enough patients treated with PARP inhibitors. Furthermore, cancer therapeutics can affect the expression of RAD51, indicating that using paired pre- and posttreatment samples could add the predictive value of RAD51. The predictive value of RAD51 should be evaluated in large-scale, multicenter prospective studies before clinical implementation.

## Conclusions

In summary, RAD51 is a pivotal gene in ovarian cancer and confers ovarian cancer dependency. Ovarian cancer has pronounced RAD51 expression compared with normal ovary. High RAD51 expression implies unfavorable survival prognosis and suggests decreased drug responsiveness in ovarian cancer patients. RAD51 has predictive value in ovarian cancer. The predictive capacity of RAD51 deserves further and deeper exploration.

## Supplementary Information

**Additional file 1: Fig. S1.** Expression profile of RAD51 in various cancers. (a) The expression of RAD51 was examined in the Human Protein Atlas. (b) Comparison of RAD51 expression between TCGA tumor samples and matched GTEx normal samples in GEPIA (one-way ANOVA). (c) Alteration frequency of RAD51 in TCGA cancers was analyzed in cBioPortal. P value was denoted as *P < 0.05.

**Additional file 2: Fig. S2.** Mutation profile of RAD51 in various cancers. (a) Mutation profile of RAD51 in TCGA cancers was analyzed in cBioPortal. (b) Integrated mutation profile of RAD51 in a collection of TCGA cancers.

**Additional file 3: Fig. S3.** Mutation profile of RAD51 in ovarian cancer. (a) The mutation profile of RAD51 in TCGA ovarian cancer was studied using cBioPortal. (b) Patients were divided into a disease-free group and a recurred/progressed group. RAD51 expression was compared between these two groups.

**Additional file 4: Fig. S4.** Gene correlations of RAD51 in ovarian cancer. According to the TCGA study published in 2011, BRCA2, CDK12, CSMD3, FAT3, GABRA6, NF1, RB1, and TP53 are highly mutated genes in ovarian cancer. (a) Correlation analysis between RAD51 and the nine genes (Pearson’s correlation test). (b) The expression of RAD51 and the nine genes was assessed in R2.

**Additional file 5: Table S1.** Genes correlated with RAD51 in UALCAN.

**Additional file 6: Table S2.** Gene Ontology pathways enriched with genes correlated with RAD51.

**Additional file 7: Table S3.** KEGG pathways enriched with genes correlated with RAD51.

**Additional file 8: Table S4.** Genes interacted with RAD51 in NCBI.

**Additional file 9: Fig. S5.** Protein interactions of RAD51. In UALCAN, 557 genes correlated with RAD51 in TCGA ovarian cancer. In NCBI, 209 genes interacted with RDA51. These 209 genes overlapped with the 557 genes correlated with RAD51, producing 36 overlapping genes. The protein–protein interaction network of RAD51 with the 36 overlapping genes was constructed.

**Additional file 10: Fig. S6.** High RAD51 expression denotes poor survival in dataset GSE26193. According to RAD51 expression, patients in the dataset GSE26193 were split into two groups. Survival analysis was performed and Kaplan–Meier survival curves of OS and PFS in (a) the general patients, (b) patients receiving platinum-containing chemotherapy, and (c) patients treated with taxol were shown (log-rank test).

**Additional file 11: Fig. S7.** High RAD51 expression denotes poor survival in dataset GSE63885. According to RAD51 expression, patients in the dataset GSE63885 were split into two groups. Survival analysis was performed and Kaplan–Meier survival curves of OS and PFS in (a) the general patients, (b) patients receiving platinum-containing chemotherapy, and (c) patients treated with taxol were shown (log-rank test).

**Additional file 12: Fig. S8.** High RAD51 expression implies elevated PARP inhibitor tolerance in ovarian cancer. In DepMap, correlation analysis between RAD51 gene effect and responsiveness of ovarian cancer cell lines to (a) olaparib, (b) niraparib, (c) rucaparib, and (d) talazoparib was performed (Pearson’s correlation test).

**Additional file 13: Fig. S9.** RAD51 affects immune infiltration in ovarian cancer. The effect of RAD51 on tumor-infiltrating immune cells in ovarian cancer was assessed using TIMER. The correlation between RAD51 expression and tumor-infiltrating B cells, T cells, hematopoietic stem cells, and MDSCs (myeloid-derived suppressor cells) was analyzed (Spearman’s correlation test).

## Data Availability

Not applicable.

## Abbreviations

DepMap: Cancer Dependency Map
GEPIA: Gene Expression Profiling Interactive Analysis
HR: Hazard ratio
OS: Overall survival
PARP: Poly-(ADP-ribose)-polymerase
PFS: Progression-free survival
