# Supplementary figures and images for: Predictive value of RAD51 on the survival and drug responsiveness of ovarian cancer
Source: Cancer Cell Int. 2021 May 5;21:249. doi: 10.1186/s12935-021-01953-5 (PMC8097773; doi:10.1186/s12935-021-01953-5)

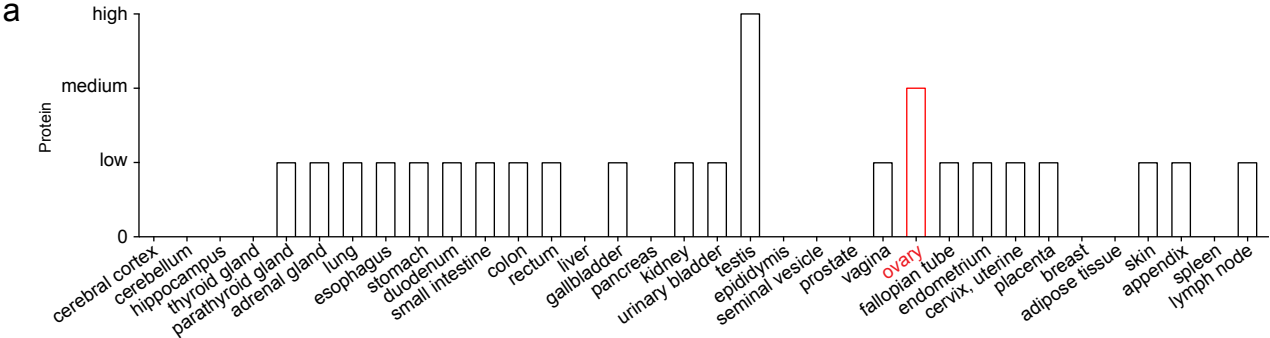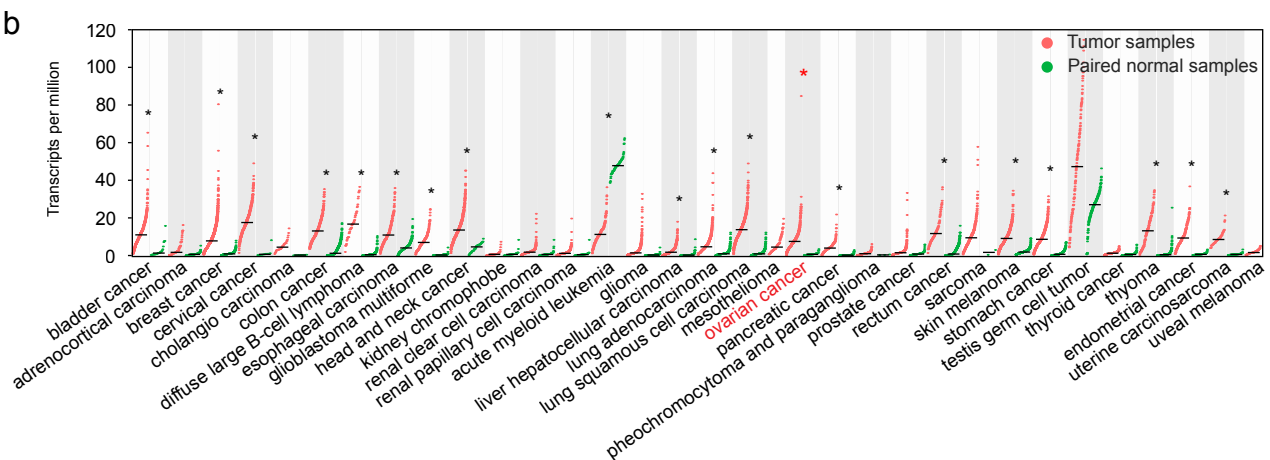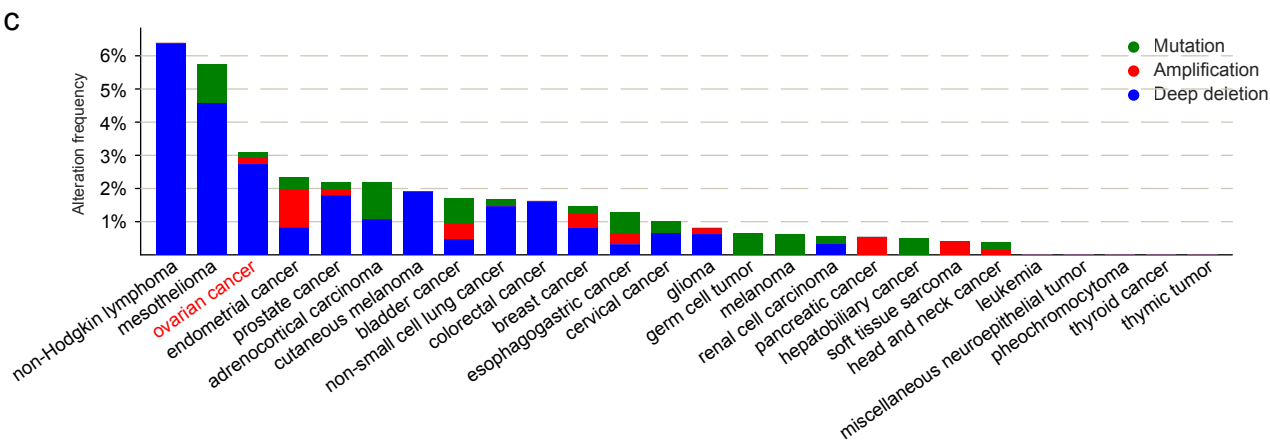

Supplement: Supplementary file 1 — Additional file 1: Fig. S1. Expression profile of RAD51 in various cancers. (a) The expression of RAD51 was examined in the Human Protein Atlas. (b) Comparison of RAD51 expression between TCGA tumor samples and matched GTEx normal samples in GEPIA (one-way ANOVA). (c) Alteration frequency of RAD51 in TCGA cancers was analyzed in cBioPortal. P value was denoted as *P < 0.05. [file 12935_2021_1953_MOESM1_ESM.pdf]

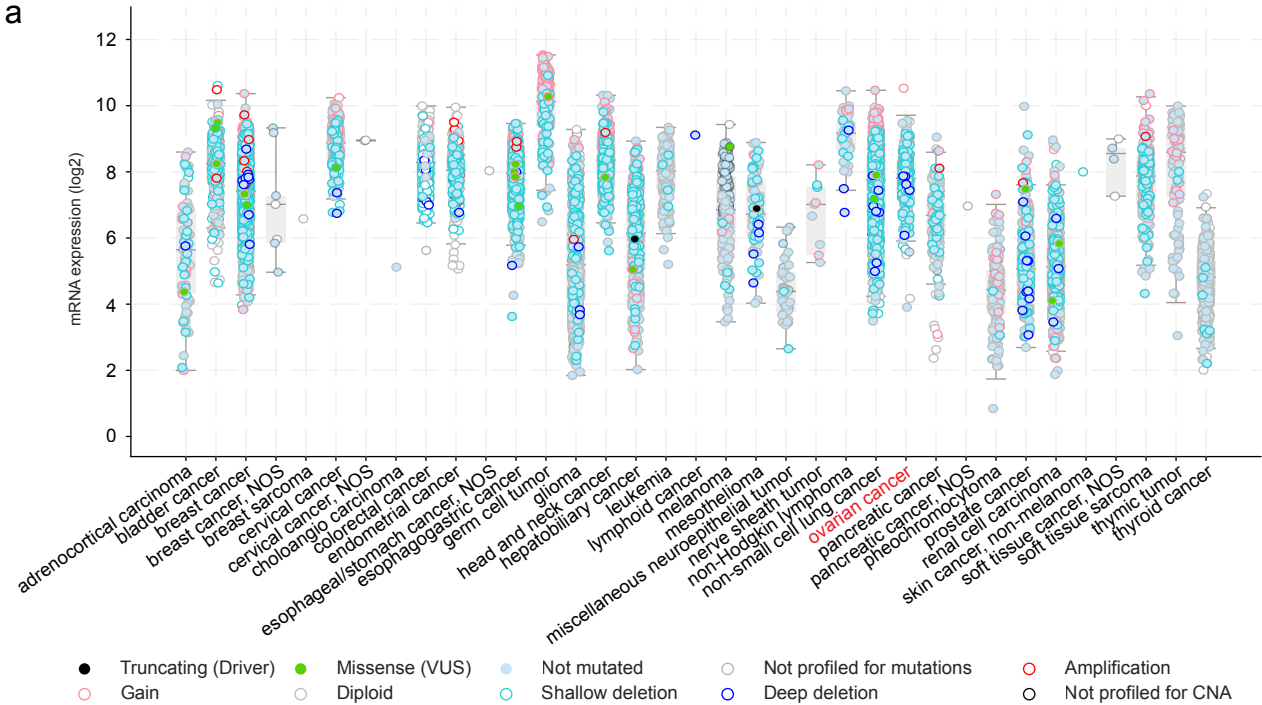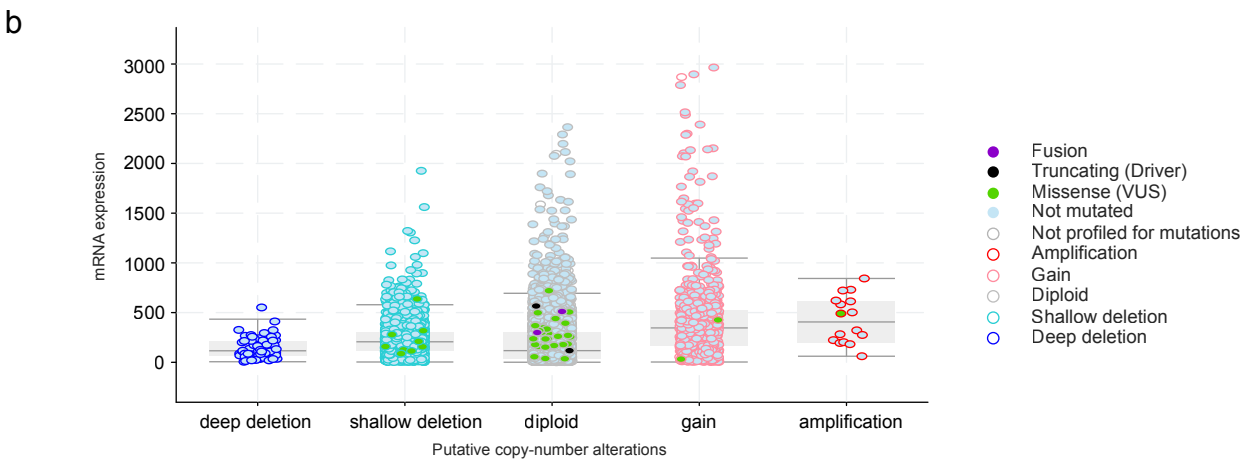

Supplement: Supplementary file 2 — Additional file 2: Fig. S2. Mutation profile of RAD51 in various cancers. (a) Mutation profile of RAD51 in TCGA cancers was analyzed in cBioPortal. (b) Integrated mutation profile of RAD51 in a collection of TCGA cancers. [file 12935_2021_1953_MOESM2_ESM.pdf]

**a**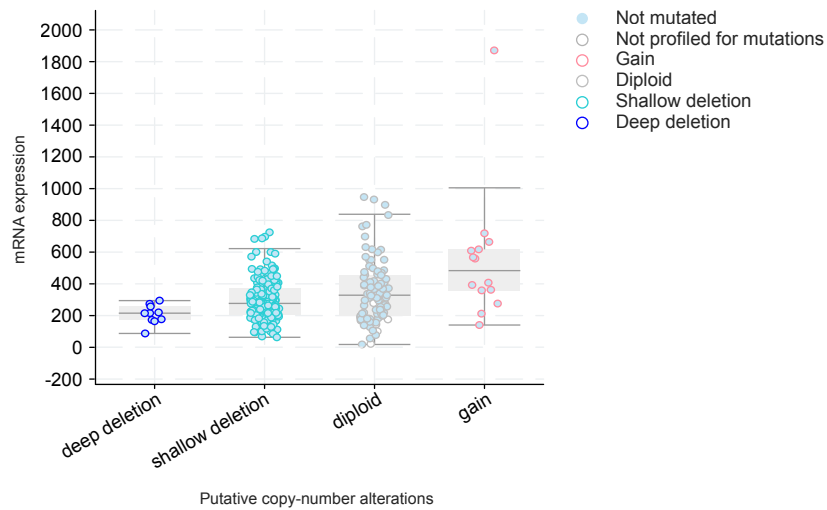**b**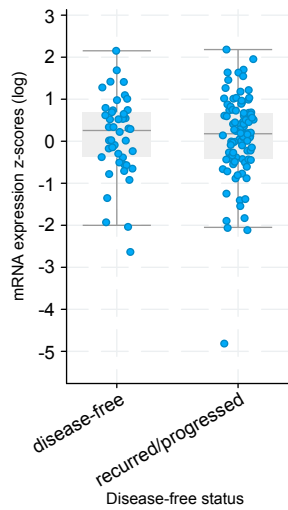

Supplement: Supplementary file 3 — Additional file 3: Fig. S3. Mutation profile of RAD51 in ovarian cancer. (a) The mutation profile of RAD51 in TCGA ovarian cancer was studied using cBioPortal. (b) Patients were divided into a disease-free group and a recurred/progressed group. RAD51 expression was compared between these two groups. [file 12935_2021_1953_MOESM3_ESM.pdf]

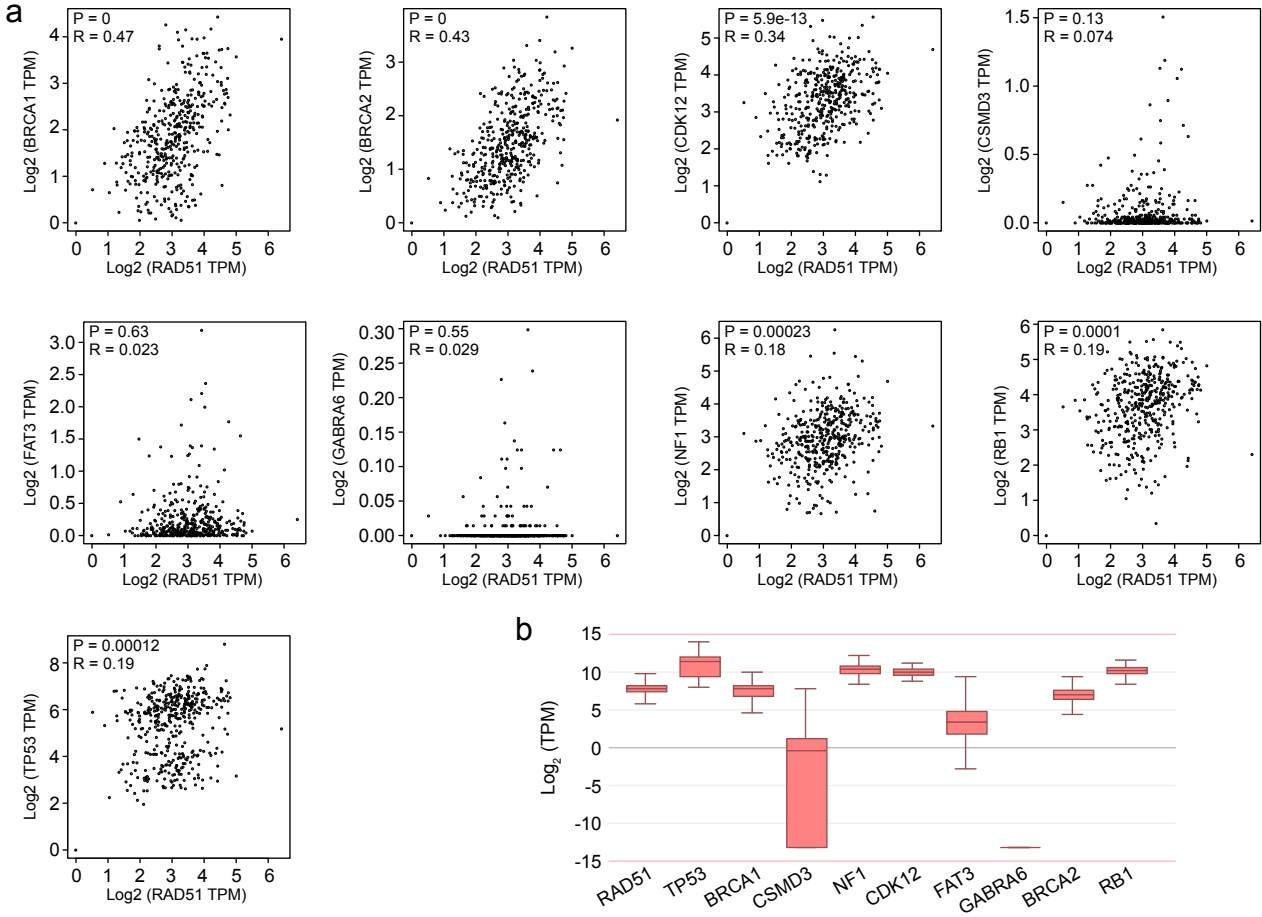

Supplement: Supplementary file 4 — Additional file 4: Fig. S4. Gene correlations of RAD51 in ovarian cancer. According to the TCGA study published in 2011, BRCA2, CDK12, CSMD3, FAT3, GABRA6, NF1, RB1, and TP53 are highly mutated genes in ovarian cancer. (a) Correlation analysis between RAD51 and the nine genes (Pearson’s correlation test). (b) The expression of RAD51 and the nine genes was assessed in R2. [file 12935_2021_1953_MOESM4_ESM.pdf]

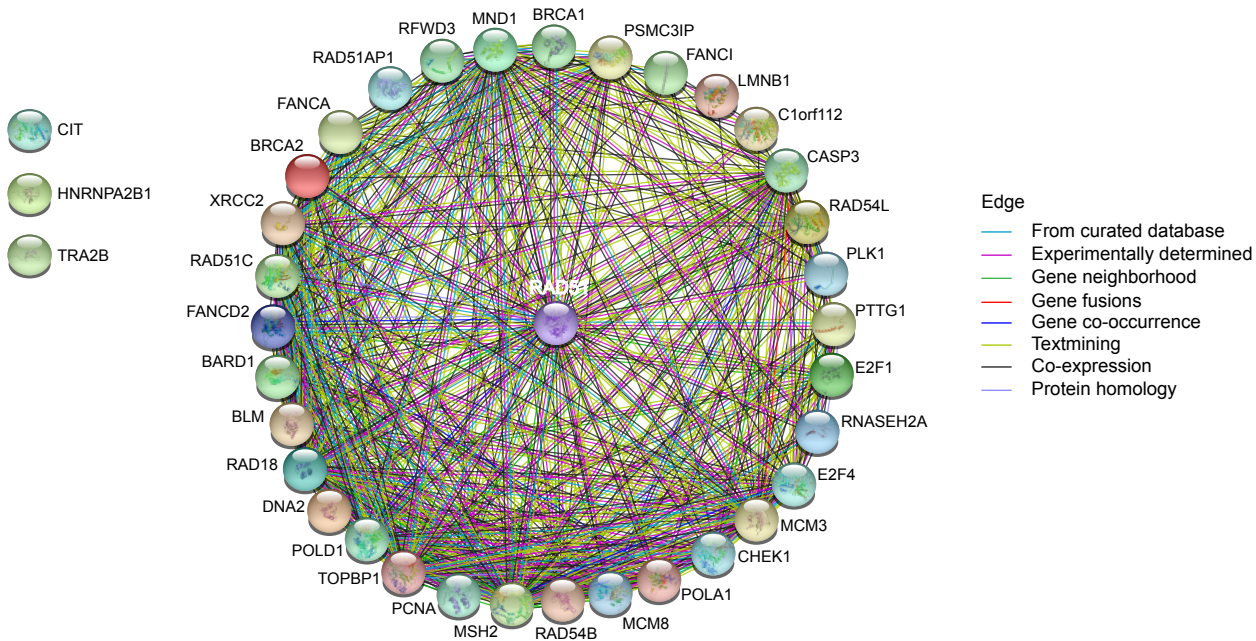

Supplement: Supplementary file 9 — Additional file 9: Fig. S5. Protein interactions of RAD51. In UALCAN, 557 genes correlated with RAD51 in TCGA ovarian cancer. In NCBI, 209 genes interacted with RDA51. These 209 genes overlapped with the 557 genes correlated with RAD51, producing 36 overlapping genes. The protein–protein interaction network of RAD51 with the 36 overlapping genes was constructed. [file 12935_2021_1953_MOESM9_ESM.pdf]

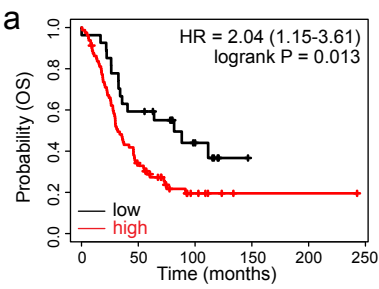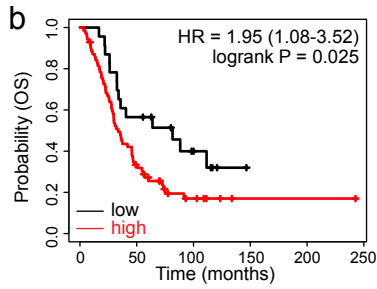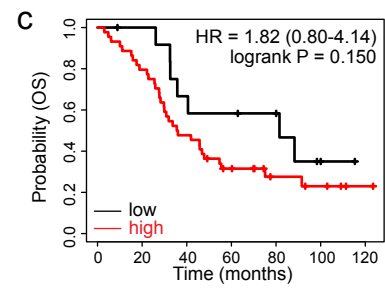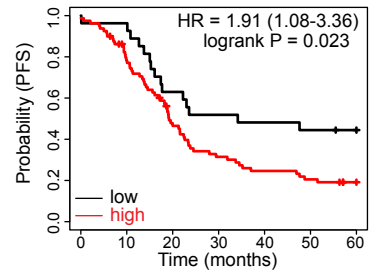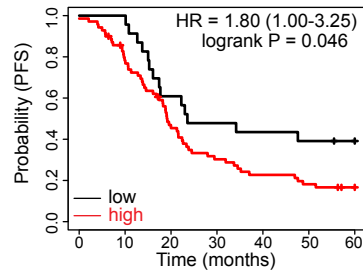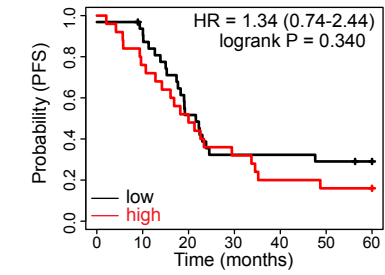

Supplement: Supplementary file 10 — Additional file 10: Fig. S6. High RAD51 expression denotes poor survival in dataset GSE26193. According to RAD51 expression, patients in the dataset GSE26193 were split into two groups. Survival analysis was performed and Kaplan–Meier survival curves of OS and PFS in (a) the general patients, (b) patients receiving platinum-containing chemotherapy, and (c) patients treated with taxol were shown (log-rank test). [file 12935_2021_1953_MOESM10_ESM.pdf]

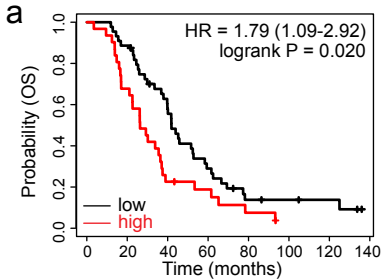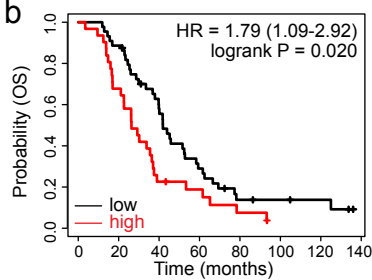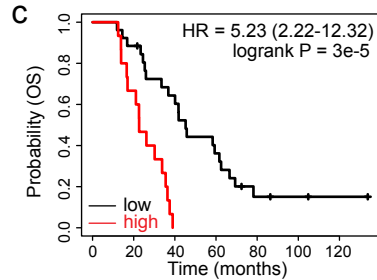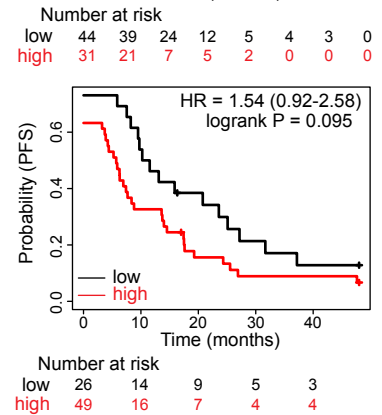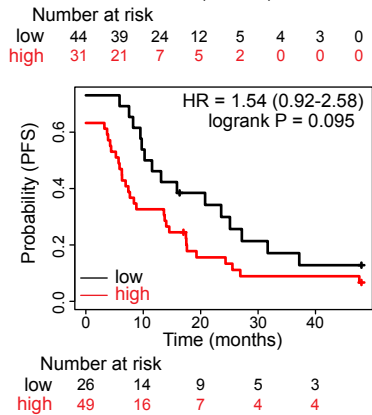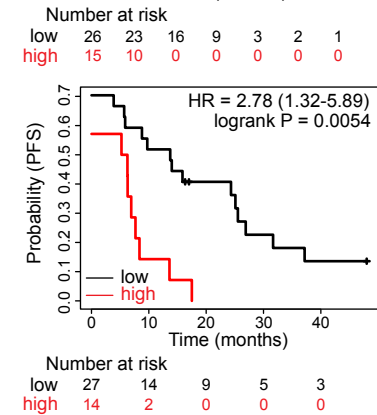

Supplement: Supplementary file 11 — Additional file 11: Fig. S7. High RAD51 expression denotes poor survival in dataset GSE63885. According to RAD51 expression, patients in the dataset GSE63885 were split into two groups. Survival analysis was performed and Kaplan–Meier survival curves of OS and PFS in (a) the general patients, (b) patients receiving platinum-containing chemotherapy, and (c) patients treated with taxol were shown (log-rank test). [file 12935_2021_1953_MOESM11_ESM.pdf]

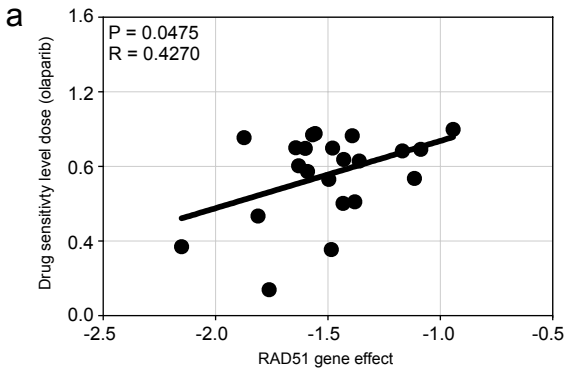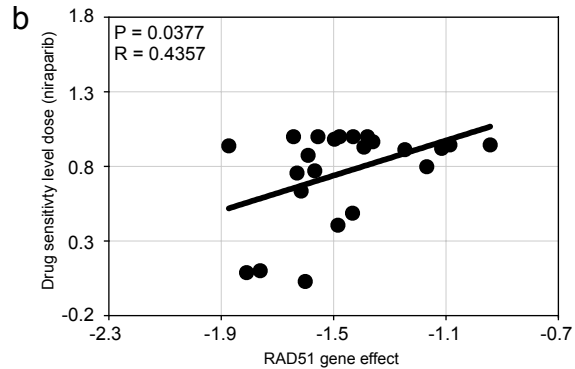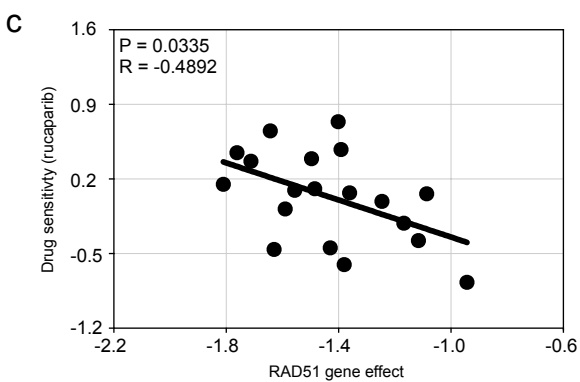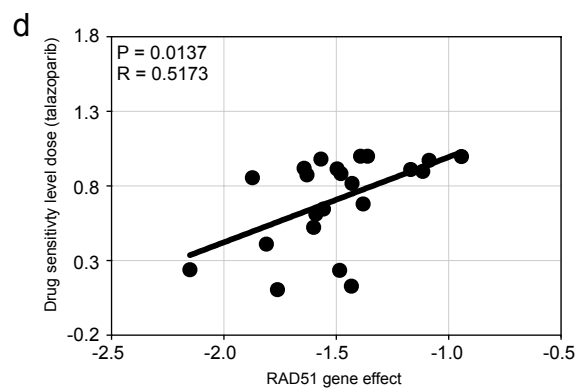

Supplement: Supplementary file 12 — Additional file 12: Fig. S8. High RAD51 expression implies elevated PARP inhibitor tolerance in ovarian cancer. In DepMap, correlation analysis between RAD51 gene effect and responsiveness of ovarian cancer cell lines to (a) olaparib, (b) niraparib, (c) rucaparib, and (d) talazoparib was performed (Pearson’s correlation test). [file 12935_2021_1953_MOESM12_ESM.pdf]

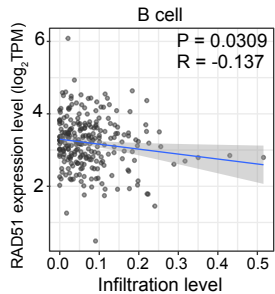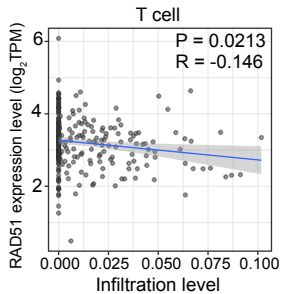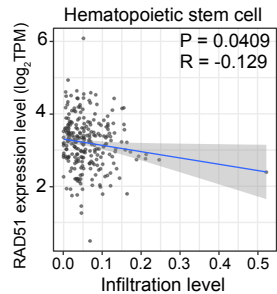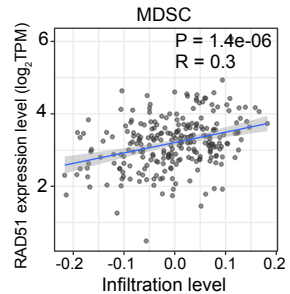

Supplement: Supplementary file 13 — Additional file 13: Fig. S9. RAD51 affects immune infiltration in ovarian cancer. The effect of RAD51 on tumor-infiltrating immune cells in ovarian cancer was assessed using TIMER. The correlation between RAD51 expression and tumor-infiltrating B cells, T cells, hematopoietic stem cells, and MDSCs (myeloid-derived suppressor cells) was analyzed (Spearman’s correlation test). [file 12935_2021_1953_MOESM13_ESM.pdf]
