# Supplementary material for: Predictive value of RAD51 on the survival and drug responsiveness of ovarian cancer
Source: Cancer Cell Int. 2021 May 5;21:249. doi: 10.1186/s12935-021-01953-5 (PMC8097773; doi:10.1186/s12935-021-01953-5)
Supplement: Supplementary file 6 — Additional file 6: Table S2. Gene Ontology pathways enriched with genes correlated with RAD51. [file 12935_2021_1953_MOESM6_ESM.docx]

| **Term** | **Database** | **ID** | **Corrected P value** |
| --- | --- | --- | --- |
| DNA repair | Gene Ontology | GO:0006281 | 5.61E-34 |
| cellular response to DNA damage stimulus | Gene Ontology | GO:0006974 | 3.42E-16 |
| interstrand cross-link repair | Gene Ontology | GO:0036297 | 1.04E-14 |
| double-strand break repair | Gene Ontology | GO:0006302 | 1.55E-14 |
| double-strand break repair via homologous recombination | Gene Ontology | GO:0000724 | 3.62E-14 |
| double-strand break repair via break-induced replication | Gene Ontology | GO:0000727 | 2.60E-12 |
| damaged DNA binding | Gene Ontology | GO:0003684 | 7.87E-12 |
| DNA damage response, signal transduction by p53 class mediator resulting in cell cycle arrest | Gene Ontology | GO:0006977 | 1.31E-10 |
| double-strand break repair via nonhomologous end joining | Gene Ontology | GO:0006303 | 1.52E-09 |
| site of double-strand break | Gene Ontology | GO:0035861 | 4.78E-08 |
| nucleotide-excision repair, DNA gap filling | Gene Ontology | GO:0006297 | 9.48E-08 |
| DNA recombination | Gene Ontology | GO:0006310 | 1.24E-07 |
| DNA damage checkpoint | Gene Ontology | GO:0000077 | 6.88E-07 |
| DNA synthesis involved in DNA repair | Gene Ontology | GO:0000731 | 1.23E-06 |
| DNA damage response, detection of DNA damage | Gene Ontology | GO:0042769 | 1.41E-06 |
| transcription-coupled nucleotide-excision repair | Gene Ontology | GO:0006283 | 1.69E-06 |
| mismatch repair | Gene Ontology | GO:0006298 | 8.55E-06 |
| Fanconi anaemia nuclear complex | Gene Ontology | GO:0043240 | 4.15E-05 |
| DNA double-strand break processing | Gene Ontology | GO:0000729 | 5.36E-05 |
| base-excision repair | Gene Ontology | GO:0006284 | 6.90E-05 |
| positive regulation of DNA repair | Gene Ontology | GO:0045739 | 8.07E-05 |
| nucleotide-excision repair, DNA incision, 5'-to lesion | Gene Ontology | GO:0006296 | 0.00015883 |
| nucleotide-excision repair, DNA incision | Gene Ontology | GO:0033683 | 0.00017765 |
| mitotic G2 DNA damage checkpoint | Gene Ontology | GO:0007095 | 0.00018009 |
| postreplication repair | Gene Ontology | GO:0006301 | 0.00031235 |
| signal transduction involved in G2 DNA damage checkpoint | Gene Ontology | GO:0072425 | 0.00031235 |
| intrinsic apoptotic signaling pathway in response to DNA damage by p53 class mediator | Gene Ontology | GO:0042771 | 0.00063667 |
| DNA damage response, signal transduction by p53 class mediator resulting in transcription of p21 class mediator | Gene Ontology | GO:0006978 | 0.00064108 |
| intra-S DNA damage checkpoint | Gene Ontology | GO:0031573 | 0.00079429 |
| base-excision repair, gap-filling | Gene Ontology | GO:0006287 | 0.00131073 |
| BRCA1-A complex | Gene Ontology | GO:0070531 | 0.00233117 |
| site of DNA damage | Gene Ontology | GO:0090734 | 0.00298 |
| positive regulation of double-strand break repair via homologous recombination | Gene Ontology | GO:1905168 | 0.00457188 |
| positive regulation of double-strand break repair | Gene Ontology | GO:2000781 | 0.00542168 |
| positive regulation of double-strand break repair via nonhomologous end joining | Gene Ontology | GO:2001034 | 0.01025076 |
| nucleotide-excision repair | Gene Ontology | GO:0006289 | 0.01276971 |
| intrinsic apoptotic signaling pathway in response to DNA damage | Gene Ontology | GO:0008630 | 0.01720576 |
| DNA ligation involved in DNA repair | Gene Ontology | GO:0051103 | 0.02600215 |
| DNA damage induced protein phosphorylation | Gene Ontology | GO:0006975 | 0.03071572 |
| mitotic G1 DNA damage checkpoint | Gene Ontology | GO:0031571 | 0.03071572 |
| nonhomologous end joining complex | Gene Ontology | GO:0070419 | 0.03071572 |
| regulation of DNA damage checkpoint | Gene Ontology | GO:2000001 | 0.03582404 |
| regulation of DNA repair | Gene Ontology | GO:0006282 | 0.04063838 |
| response to drug | Gene Ontology | GO:0042493 | 0.04896619 |

**Table S2.** Gene Ontology pathways enriched with genes correlated with RAD51.
