# Supplementary material for: Predictive value of RAD51 on the survival and drug responsiveness of ovarian cancer
Source: Cancer Cell Int. 2021 May 5;21:249. doi: 10.1186/s12935-021-01953-5 (PMC8097773; doi:10.1186/s12935-021-01953-5)
Supplement: Supplementary file 7 — Additional file 7: Table S3. KEGG pathways enriched with genes correlated with RAD51. [file 12935_2021_1953_MOESM7_ESM.docx]

| **Term** | **Database** | **ID** | **corrected P value** |
| --- | --- | --- | --- |
| Fanconi anemia pathway | KEGG PATHWAY | hsa03460 | 1.68E-15 |
| Homologous recombination | KEGG PATHWAY | hsa03440 | 3.13E-13 |
| Mismatch repair | KEGG PATHWAY | hsa03430 | 5.04E-09 |
| Base excision repair | KEGG PATHWAY | hsa03410 | 6.18E-08 |
| Nucleotide excision repair | KEGG PATHWAY | hsa03420 | 7.10E-07 |
| Non-homologous end-joining | KEGG PATHWAY | hsa03450 | 0.00646419 |
| Platinum drug resistance | KEGG PATHWAY | hsa01524 | 0.01743103 |
| Drug metabolism - other enzymes | KEGG PATHWAY | hsa00983 | 0.02221219 |

**Table S3.** KEGG pathways enriched with genes correlated with RAD51.
